# Clipping has stronger effects on plant production than does warming in three alpine meadow sites on the Northern Tibetan Plateau

**DOI:** 10.1038/s41598-017-16645-2

**Published:** 2017-11-27

**Authors:** Gang Fu, Zhen Xi Shen

**Affiliations:** Lhasa Plateau Ecosystem Research Station, Key Laboratory of Ecosystem Network Observation and Modeling, Institute of Geographic Sciences and Natural Resources Research, Chinese Academy of Sciences, Beijing, 100101 China

## Abstract

The relative effects of warming and clipping on vegetation growth are not fully understood. Therefore, we compared the relative effects of experimental warming and clipping on the normalised difference vegetation index (NDVI), green NDVI (GNDVI), soil-adjusted vegetation index (SAVI), aboveground biomass (AGB) and gross primary production (GPP) in three alpine meadow sites (A, B and C) on the Northern Tibetan Plateau from 2013 to 2015. There were no obvious effects of experimental warming on the NDVI, GNDVI, SAVI, AGB and GPP at the three sites, which were most likely attributed to experimental warming-induced warming and drying conditions. In contrast, clipping significantly decreased the NDVI, SAVI and AGB by 27.8%, 31.3% and 18.2% at site A, by 27.1%, 31.8% and 27.7% at site B, and by 12.3%, 15.1% and 17.6% at site C, respectively. Clipping also significantly reduced the GNDVI and GPP by 11.1% and 28.2% at site A and by 18.9% and 33.7% at site B, respectively. Clipping marginally decreased the GNDVI by 8.7% (*p* = 0.060) and GPP (*p* = 0.082) by 14.4% at site C. Therefore, clipping had stronger effects on vegetation growth than did warming in the three alpine meadow sites on the Tibetan Plateau.

## Introduction

Various vegetation indices (e.g., normalised difference vegetation index, NDVI; green NDVI, GNDVI; soil-adjusted vegetation index, SAVI) have been treated as indicators of vegetation biophysical variables (e.g., coverage, biomass, productivity and phenology)^1–5^. Aboveground biomass (AGB) can not only affect ecosystem function but also livestock carrying capacity and animal husbandry development^6,7^. Gross primary production (GPP) is vital for sequestering carbon in terrestrial ecosystems^8,9^. The NDVI, GNDVI, SAVI, AGB and GPP are all related to the global carbon cycle of terrestrial ecosystems, and understanding their responses to global change is very important for predicting future changes in vegetation productivity. Human activities and climatic change are the main driving forces in the global carbon cycle of terrestrial ecosystems^10–13^. A growing number of studies have used productivity models to investigate the relative effects of human activities and climatic change on carbon pools^14–17^. In these previous studies, the effects of warming and precipitation changes on vegetation productivity are mixed. Clipping/grazing is one of the most common modes of human disturbance and land-use types in grassland ecosystems^18–20^. Field warming and clipping/grazing experiments have been conducted to distinguish the effects of warming and clipping/grazing on vegetation productivity^21,22^. However, the relative effects of warming and clipping/grazing on vegetation productivity are still debated.

The Tibetan Plateau is one of the most sensitive regions to human activities and climatic change^10,23,24^. Almost three quarters of the surface of the Tibetan Plateau is covered with various grassland ecosystems^25,26^. These various grasslands are critical pastures and play vital roles in regulating ecological structures and functions on the Tibetan Plateau^25,27^. Alpine meadow, covering approximately one-third of the plateau area, is one of the alpine grassland types most sensitive to global change^27,28^. The surface temperature on the Tibetan Plateau has been increasing at a much greater rate than the global average^23,29^. Clipping, as a common land-use type, is often used to mimic grazing and agricultural hay harvest in alpine meadows on the Tibetan Plateau^30,31^. Warming and clipping/grazing can indirectly affect moisture regimes, and changing moisture regimes can significantly impact the recent change in the carbon cycle sensitivity to temperature variability^32,33^. Several studies have investigated the responses of aboveground biomass to warming and clipping/grazing under controlled warming and clipping/grazing conditions, but there are still inconsistent results in alpine meadows on the Tibetan Plateau^21,31,34,35^. Some studies found that experimental warming rather than clipping/grazing had significant effects on AGB across all the observed years in alpine meadows on the Tibetan Plateau^31,35^. In contrast, other studies demonstrated that clipping/grazing rather than experimental warming had significant effects on AGB across all the observed years in alpine meadows on the Tibetan Plateau^34^. These results demonstrate that it is still unclear which one of these two driving factors (warming and clipping/grazing) has a stronger effect on vegetation productivity in alpine meadows on the Tibetan Plateau.

Experimental warming and clipping/grazing have been shown to have lag effects on AGB^34,36^, and in some studies, the effects of experimental warming and clipping/grazing on AGB varied with year in alpine meadows on the Tibetan Plateau^31,35,37,38^. These findings suggest that the relative strengths of experimental warming and clipping/grazing effects on biomass production can vary over time. However, most of these warming and clipping/grazing experiments in alpine meadows on the Tibetan Plateau lasted less than 5 years^31,34,35^. Therefore, it is unclear whether the relative strengths of experimental warming and clipping/grazing effects on vegetation productivity in the short term (<5 years) are different from those in the long term (>5 years) in alpine meadows on the Tibetan Plateau.

Although these previous studies have examined the relative strengths of warming and clipping/grazing effects on AGB and GPP, no studies have investigated the relative responses of the NDVI, GNDVI and SAVI to experimental warming and clipping/grazing under controlled experimental warming and clipping/grazing conditions in alpine meadows on the Tibetan Plateau^31,34,35^. Therefore, in this study, a field warming and clipping experiment was conducted in 2008 at three alpine meadow sites on the Northern Tibetan Plateau. The monthly NDVI, GNDVI, SAVI, GPP and AGB from 2013 to 2015 were measured. The main objective of this study was to compare the relative strengths of experimental warming and clipping effects on the NDVI, GNDVI, SAVI, AGB and GPP in three alpine meadow sites on the Northern Tibetan Plateau.

## Results

### Effects of warming and clipping on soil temperature (T_s_), soil moisture (SM), air temperature (T_a_) and vapor pressure deficit (VPD)

Across all three growing seasons from 2013 to 2015, there were no significant main effects of clipping and no significant interactive effects of warming and clipping on *T*_*s*_ and SM at sites A, B and C (Table S1). Experimental warming significantly increased *T*_*s*_ by 1.31 °C, 1.27 °C and 1.17 °C but significantly decreased SM by 0.02 m^3^ m^−3^, 0.03 m^3^ m^−3^ and 0.04 m^3^ m^−3^ at sites A, B and C, respectively (Figure S1). Experimental warming significantly increased *T*_*a*_ by 1.42 °C, 1.18 °C and 1.24 °C and VPD by 0.12 kPa, 0.10 kPa and 0.08 kPa at sites A, B and C, respectively (Figure S1). Clipping significantly decreased *T*_*a*_ by 0.14 °C at site A but increased *T*_*a*_ by 0.25 °C at site C (Figure S1). Clipping significantly increased VPD by 0.02 kPa at site B.

### Effects of warming and clipping on NDVI, GNDVI, SAVI, AGB and GPP

**Figure 1:**
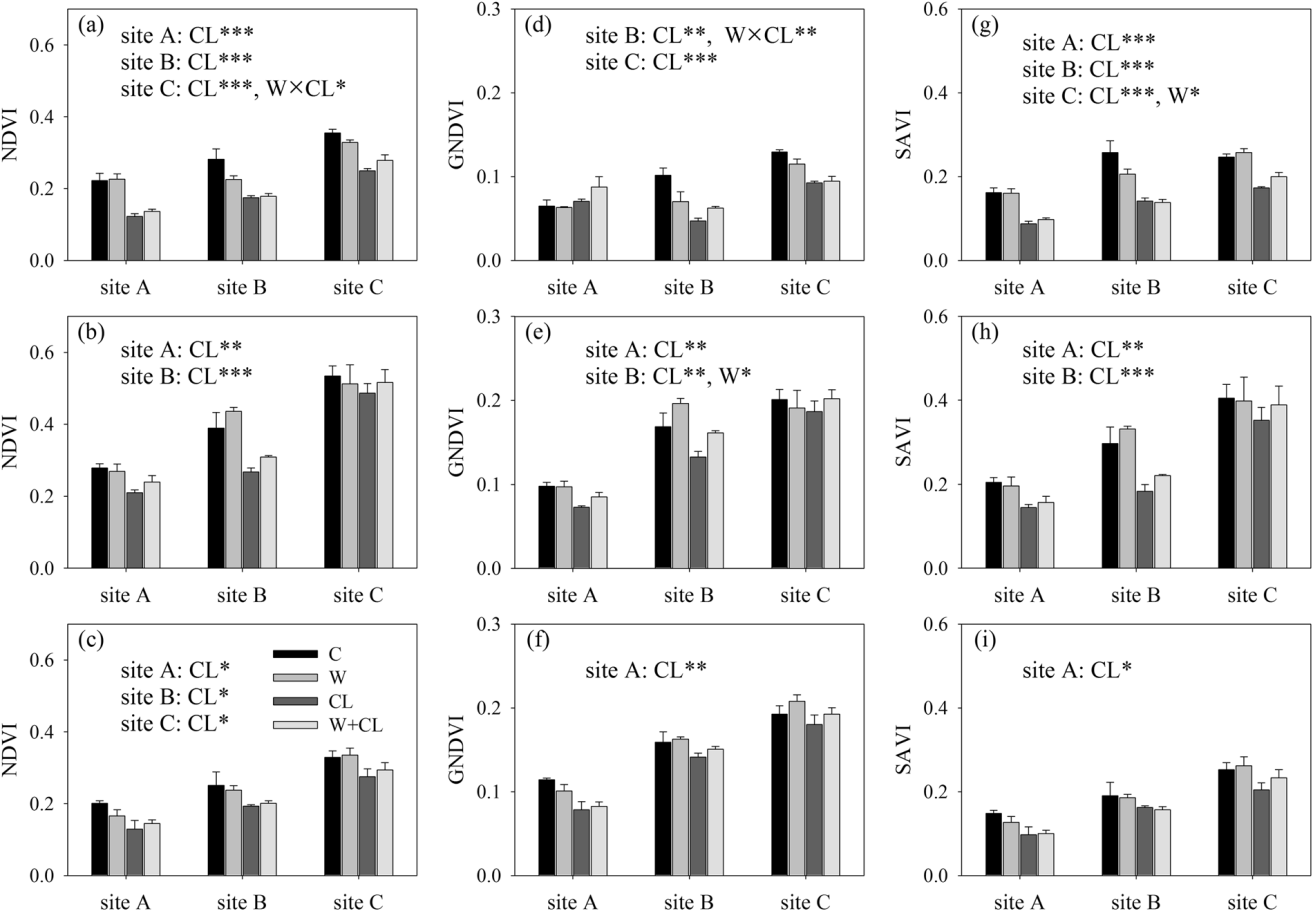
Responses of the normalised difference vegetation index (NDVI), green NDVI (GNDVI) and soil-adjusted vegetation index (SAVI) to experimental warming and clipping in (**a**,**d**,**g**) 2013, (**b**,**e**,**h**) 2014 and (**c**,**f**,**i**) 2015 in alpine meadows at sites A, B and C on the Tibetan Plateau. *, ** and ***indicate *p* < 0.05, *p* < 0.01 and *p* < 0.001, respectively.

**Figure 2:**
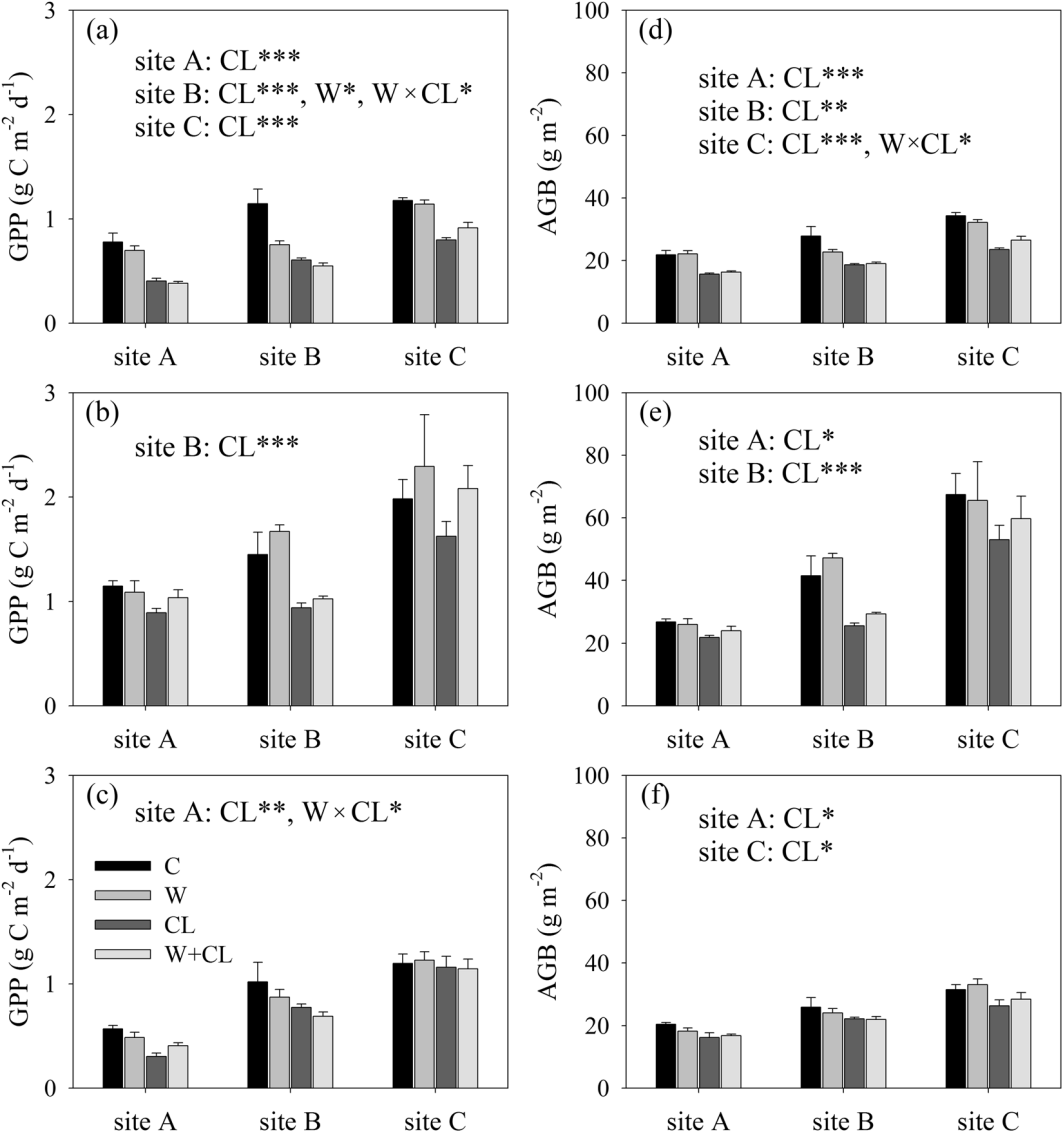
Responses of gross primary production (GPP) and aboveground biomass (AGB) to experimental warming and clipping in (**a**,**d**) 2013, (**b**,**e**) 2014 and (**c**,**f**) 2015 in alpine meadows at sites A, B and C on the Tibetan Plateau. *^,^ ** and ***indicate *p* < 0.05, *p* < 0.01 and *p* < 0.001, respectively.

There were significant inter-annual variations in the NDVI, GNDVI, SAVI, AGB and GPP (Table 1, Figs 1 and 2). The NDVI, SAVI and AGB values were obviously greater in 2014 than in 2013 and 2015, although there were no significant differences between the years 2013 and 2015 at sites A, B and C. The GNDVI was significantly lower in 2013 than in 2014 and 2015 at sites A, B and C, but there was no significant difference between the years 2014 and 2015 at sites A and C. The GPP was lowest in 2015 and highest in 2014 among the three growing seasons at site A. The GPP was lowest in 2013 and highest in 2014 among the three growing seasons at site C. Although there was no significant difference in GPP between 2013 and 2015, both years had significantly lower GPP values than 2014 at site B.

**Table 1:** Repeated-measures analysis of variance was used to estimate the main and interactive effects of experimental warming (W), clipping (CL) and measuring year (Y) on the normalised difference vegetation index (NDVI), soil adjusted vegetation index (SAVI), green NDVI (GNDVI), aboveground biomass (AGB) and gross primary production (GPP) in the alpine meadow sites A, B and C on the Tibetan Plateau.

Site	Model	NDVI	SAVI	GNDVI	AGB	GPP
A	W	0.14	0.02	0.50	0.03	0.00
	CL	49.74***	40.14***	5.64*	40.42***	44.81***
	Y	41.95***	29.78***	15.22***	46.90***	138.98***
	W × CL	3.30	1.32	3.63	2.36	5.00*
	W × Y	0.61	0.41	1.24	0.58	0.81
	CL × Y	3.49*	1.74	13.75***	2.47	3.86*
	W × CL × Y	0.51	0.09	0.06	0.45	0.52
B	W	0.25	0.02	1.85	0.15	1.65
	CL	70.07***	93.36***	17.17**	56.27***	62.26***
	Y	51.52***	20.67***	324.81***	37.25***	27.04***
	W × CL	1.54	1.07	1.82	0.56	0.83
	W × Y	2.78	2.57	10.15***	2.28	3.41*
	CL × Y	3.32	4.60*	3.65*	8.43**	2.96
	W × CL × Y	0.62	0.45	4.91*	0.54	1.28
C	W	0.12	0.97	0.23	0.29	1.61
	CL	8.80*	6.33*	4.30	7.05*	3.61
	Y	105.85***	52.86***	118.75***	58.50***	37.89***
	W × CL	1.47	0.54	0.82	0.65	0.14
	W × Y	0.06	0.01	1.20	0.05	1.48
	CL × Y	1.44	0.53	2.20	0.30	0.63
	W × CL × Y	0.28	0.09	0.62	0.18	0.11

*^,^ **and ***ndicate *p* < 0.05, *p* < 0.01 and *p* < 0.001, respectively.

Across all three growing seasons from 2013 to 2015, there were no significant main effects of experimental warming and no significant interactive effects of experimental warming and clipping on the NDVI, GNDVI, SAVI and AGB at sites A, B and C (Table 1, Figs 1 and 2). Experimental warming also did not significantly affect GPP at sites A, B and C (Table 1, Fig. 2). There were also no significant interactive effects of experimental warming and clipping on GPP at sites A and B (Table 1).

However, there were significant main effects of clipping (Table 1, Figs 1 and 2). In detail, clipping significantly decreased the NDVI by 27.8% (−0.06), 27.1% (−0.08) and 12.3% (−0.05) at sites A, B and C, respectively. Clipping significantly decreased the SAVI by 31.3% (−0.05), 31.8% (−0.08) and 15.1% (−0.05) at sites A, B and C, respectively. Clipping significantly decreased AGB by 18.2% (−4.11 g m^−2^), 27.7% (−8.73 g m^−2^) and 17.6% (−7.75 g m^−2^) at sites A, B and C, respectively. Clipping significantly decreased the GNDVI by 11.1% (−0.01) and 18.9% (−0.03) at sites A and B, respectively. Clipping significantly decreased GPP by 28.2% (−0.22 g C m^−2^ d^−1^) and 33.7% (−0.39 g C m^−2^ d^−1^) at sites A and B, respectively. Clipping marginally reduced the GNDVI by 8.7% (−0.02) (*p* = 0.06) and GPP by 14.4% (−0.22 g C m^−2^ d^−1^) (*p* = 0.08) at site C.

### Relationships between the NDVI, GNDVI, SAVI, AGB and GPP and T_s_, SM, T_a_ and VPD

**Figure 3:**
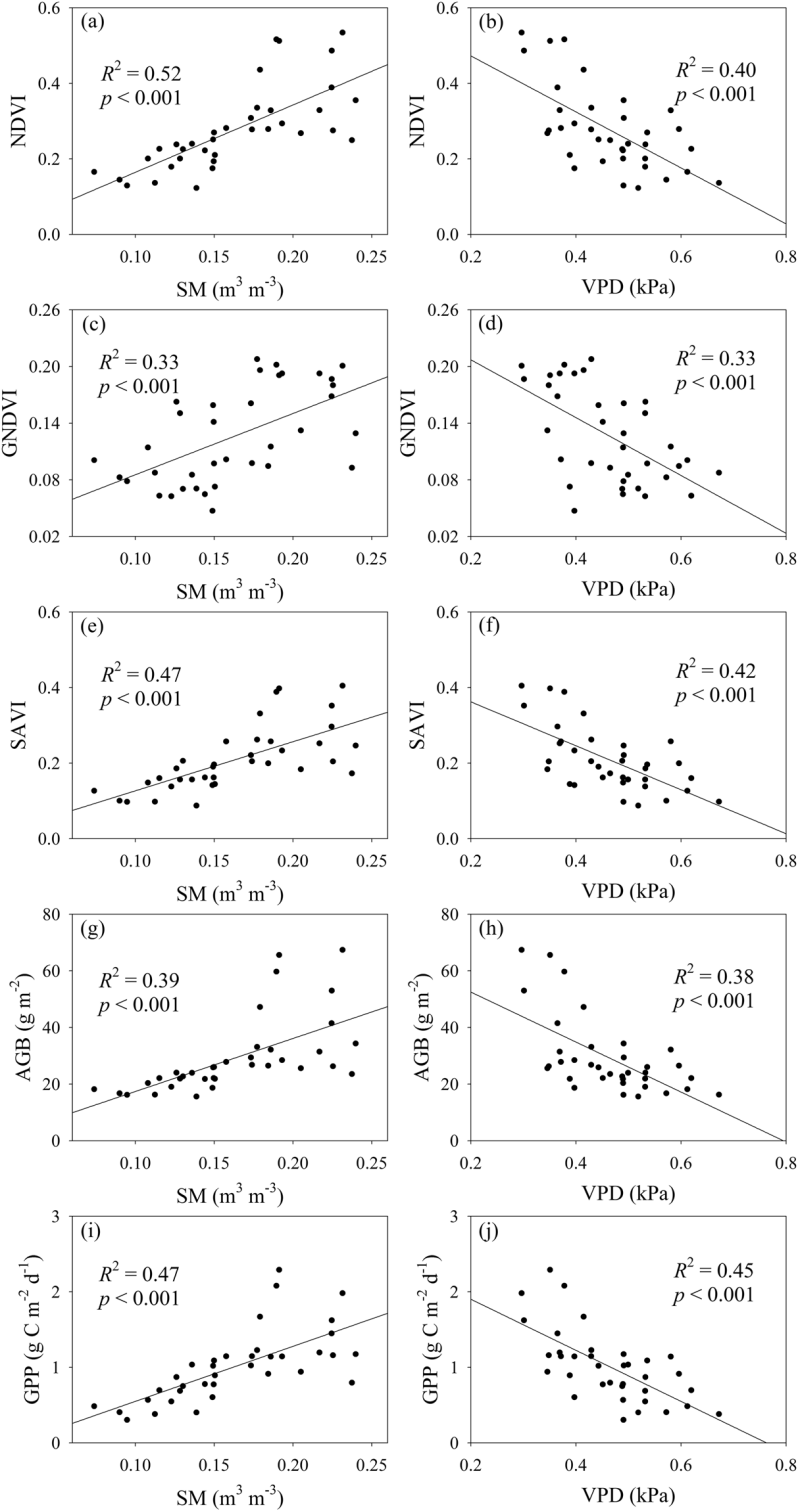
Relationships (**a**) between the normalised difference vegetation index (NDVI) and soil moisture (SM), (**b**) between the NDVI and vapor pressure deficit (VPD), (**c**) between the green NDVI (GNDVI) and SM, (**d**) between the GNDVI and VPD, (**e**) between the soil-adjusted vegetation index (SAVI) and SM, (**f**) between the SAVI and VPD, (**g**) between aboveground biomass (AGB) and SM, (**h**) between AGB and VPD, (**i**) between gross primary production (GPP) and SM, and (**j**) between GPP and VPD in alpine meadows on the Tibetan Plateau.

**Figure 4:**
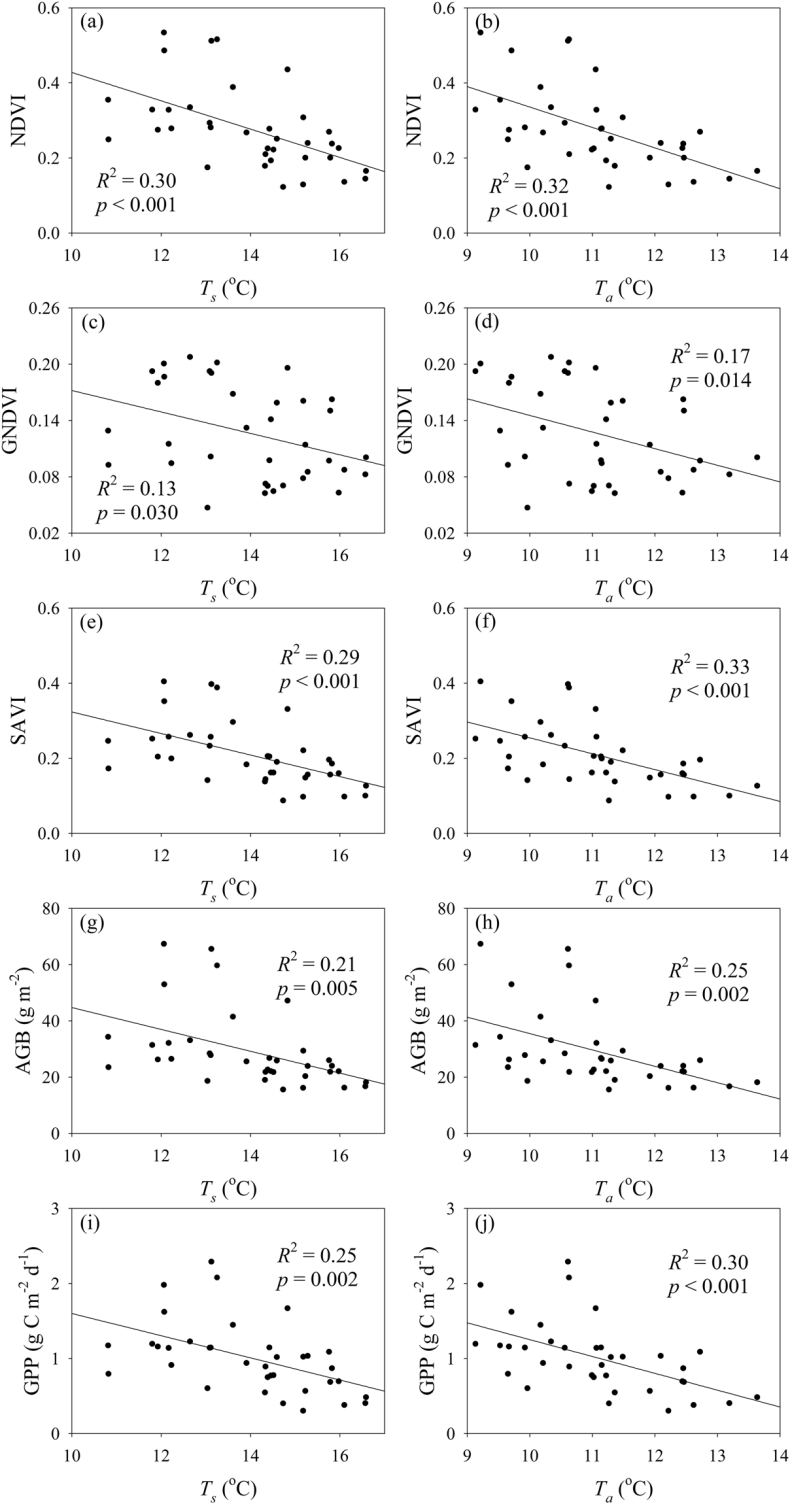
Relationships (**a**) between the normalised difference vegetation index (NDVI) and soil temperature (*T*_s_), (**b**) between the NDVI and air temperature (*T*_a_), (**c**) between the green NDVI (GNDVI) and *T*_s_, (**d**) between the GNDVI and *T*_a_, (**e**) between the soil-adjusted vegetation index (SAVI) and *T*_s_, (**f**) between the SAVI and *T*_a_, (**g**) between aboveground biomass (AGB) and *T*_s_, (**h**) between AGB and *T*_a_, (**i**) between gross primary production (GPP) and *T*_s_, and (**j**) between GPP and *T*_a_ in alpine meadows on the Tibetan Plateau.

*T*_s_, SM, *T*_a_ and VPD significantly explained 30%, 52%, 32% and 40% of the variation in the NDVI, respectively (Figs 3 and 4). *T*_*s*_, SM, *T*_*a*_ and VPD significantly explained 13%, 33%, 17% and 33% of the variation in the GNDVI, respectively (Figs 3 and 4). *T*_*s*_, SM, *T*_*a*_ and VPD significantly explained 29%, 47%, 33% and 42% of the variation in the SAVI, respectively (Figs 3 and 4). *T*_*s*_, SM, *T*_*a*_ and VPD significantly explained 21%, 39%, 25% and 38% of the variation in AGB, respectively (Figs 3 and 4). *T*_*s*_, SM, *T*_*a*_ and VPD significantly explained 25%, 47%, 30% and 45% of the variation in GPP, respectively (Figs 3 and 4).

## Discussion

### Experimental warming effects

The impacts of water availability and warming duration on the responses of vegetation indices, AGB and GPP, to warming should be interacted, and, it can be difficult to separate their influences. Experimental warming significantly increased the GNDVI by 19.3% and marginally increased the NDVI by 13.4% only in 2014 at site B (Fig. 1). Our previous studies found that field experimental warming (applied since 2010) significantly decreased AGB and GPP in 2012 and 2015 but not 2014 near our study alpine meadow site A^39,40^. These findings were quite consistent with the interannual variations in growing season precipitation. In detail, compared to the mean growing season precipitation from 1963 to 2015 (398.7 mm), the growing season precipitation was increased in 2014 (437.3 mm) and decreased in 2012 (312.8 mm), 2013 (344.7 mm) and 2015 (300.2 mm)^41^. Likewise, experimental warming significantly increased GPP in 2012 (wet year) but not 2013 (dry year) in an alpine meadow in the region of the Yangtze River source on the Tibetan Plateau^34^. Experimental warming significantly increased GPP in 2012 and 2014 (wet year) but not 2013 (dry year) in an alpine meadow in Naqu county, Tibet Autonomous Region, China^38^. Experimental warming increased AGB in a wet alpine meadow site but decreased AGB in a dry alpine meadow site on the Tibetan Plateau^42^. Therefore, water availability regulates the responses of biomass and productivity to warming in alpine meadows on the Tibetan Plateau^39^. This finding may be attributed to the following mechanisms. First, nitrogen addition can increase grassland vegetation growth^43^, and moisture availability can regulate the warming effects on soil nitrogen availability in alpine meadows on the Tibetan Plateau^44^. Second, the minimum value of soil moisture is 11.8% for meadow vegetation growth^45^, and warming-induced soil drying may aggravate the negative effects of low soil moisture on alpine vegetation growth^39^. Third, warming-induced increases in VPD may cause stomatal closure, affect stomatal conductance and intercellular CO_2_ concentration, and depress vegetation photosynthesis^46^.

There were inconsistent interannual variations in growing season precipitation and productivity in the current study. Experimental warming significantly reduced AGB and GPP in 2012 but not 2013, 2014 and 2015 at site A^41^; significantly reduced GPP in 2013 but not 2012, 2014 and 2015 at site B; and significantly increased the SAVI in 2013 but not 2012, 2014 and 2015 at site C (Figs 1 and 2). Likewise, experimental warming did not significantly alter AGB in 1998, but it significantly reduced AGB in 1999–2001 in an alpine meadow at the Haibei station on the Tibetan Plateau^31^. Experimental warming significantly reduced AGB in 2009 but not 2007 and 2008 in an alpine meadow in Hongyuan county on the Tibetan Plateau^36^. These findings suggest that warming may have legacy effects on biomass production in alpine meadows on the Tibetan Plateau^31,36^. The close relationship between warming duration and warming effects on vegetation biomass and production may be related to the following mechanisms. First, plant photosynthetic capacity is generally positively related to leaf nitrogen content^47,48^, and the positive effect of warming on the leaf nitrogen concentration can decrease with warming duration^49^. Second, soil biota can regulate soil nitrogen availability^44^, which in turn can limit alpine vegetation growth^43^. The effects of warming on the abundance of soil biota are reduced with warming duration^50^.

### Clipping effects

Our findings suggested that clipping significantly or marginally significantly reduced vegetation biomass and production across all three growing seasons in 2013–2015 (Table 1, Figs 1 and 2). Our previous study, which was conducted in the same alpine meadow sites, demonstrated that clipping significantly reduced AGB and GPP in 2012^41^. Similarly, heavy grazing resulted in a decrease in AGB and caused degradation from 2006 to 2010 in an alpine meadow in the Haibei region^35^. Clipping also significantly reduced AGB in an alpine meadow in the Yangtze River Source Region^34^. However, clipping marginally (*p* < 0.10) increased AGB from 1998 to 2001 in an alpine meadow in the Haibei region^31^. These different responses of vegetation biomass and production to clipping/grazing can be attributed to clipping intensity and frequency. For example, clipping occurred three times during the growing season, and on average, approximately 43%, 44% and 47% of the total maximum aboveground biomass was removed in the alpine meadow sites A, B and C, respectively. Grazing occurred two or three times during the growing season, and an average of approximately 50% of the total maximum aboveground biomass was removed in Wang *et al*.^35^. An average of approximately 37% of the total maximum aboveground biomass was removed in Peng *et al*.^34^. In contrast, clipping occurred only once a year and 15% of the total maximum aboveground biomass was removed in Klein *et al*.^31^.

The effects of clipping on vegetation biomass and production may be attributed to the following mechanisms. First, clipping can cause compensatory growth, and the compensatory growth magnitudes may decrease with clipping intensity^51,52^. Second, clipping can reduce green leaf area and fractional photosynthetically active radiation absorbed by vegetation^51,53^, which in turn can suppress vegetation photosynthesis and production accumulation. Third, clipping may also affect vegetation growth by indirectly influencing environmental temperature and moisture conditions. However, clipping only significantly affected *T*_*a*_ by 0.14 °C at site A and by 0.25 °C at site C. Clipping only significantly increased VPD by 0.02 kPa at site B but not sites A and C. Clipping did not affect *T*_*s*_ and SM at the three alpine meadow sites (Figs 3 and 4). Moreover, SM had the strongest effects on vegetation indices, AGB and GPP (Figs 3 and 4). These findings suggest that the clipping-induced changes in environmental temperature and moisture conditions most likely have negligible effects on vegetation biomass and production in the three alpine meadow sites on the Northern Tibetan Plateau.

### Stronger effects of clipping than experimental warming

Our findings suggested that the response magnitudes of the NDVI, GNDVI, SAVI, AGB and GPP to clipping were stronger than those to experimental warming (Table 1). Our previous study, which was conducted in the same alpine meadow sites, also implied that clipping had stronger effects on GPP and AGB in 2012 than did experimental warming^41^. Similarly, clipping had stronger effects on AGB than did experimental warming in an alpine meadow in the Yangtze River Source Region, inland of the Tibetan Plateau^34^. This finding was most likely attributed to the following mechanisms. The relatively small effects of experimental warming on vegetation biomass and production can be related to experimental warming-induced soil drying. The growing season precipitation across the three consecutive growing seasons in 2013–2015 was approximately 9.52% lower than the mean growing season precipitation in 1963–2015 (398.7 mm). This dry condition can further dampen the warming effects on vegetation growth and generally result in negligible effects of warming on vegetation biomass and production. In contrast, clipping may significantly decrease leaf area and plant photosynthesis. This mechanism explained the relatively large effects of clipping on vegetation biomass and production.

## Methods

### Study Area and Experimental Design

The study area was located at the Damxung Grassland Observation Station, Tibet Autonomous Region in China. The growing season (June–September) mean air temperature is 10.0 °C, and the growing season total precipitation is 398.7 mm based on the Damxung County meteorological stations in 1963–2015^39^. This study was conducted in three alpine meadow sites (site A: 30 °30′N, 91 °04′E; site B: 30 °31′N, 91 °04′E; site C: 30 °32′N, 91 °03′E) on a south-facing slope on the Nyainqentanglha Mountains. The dominant species at sites A and B are *Stipa capillata*, *Carex montis-everestii* and *Kobresia pygmaea*, and the dominant species at site C is *Kobresia pygmaea*. Soil organic carbon at 0–30 cm depth is 19.83, 24.04 and 43.74 g kg^−1^ at sites A, B and C, respectively. Soil total nitrogen at 0–30 cm depth is 2.12, 2.18 and 3.32 g kg^−1^ at sites A, B and C, respectively.

At each site, the field experiment was based on a complete two-by-two factorial design (warming and clipping) with three replicates of four treatments: control plots (C), warmed plots (W), clipped plots (CL), and warmed plus clipped plots (W + CL). Open top chambers (OTCs, with a bottom diameter of 1.45 m, a top diameter of 1.00 m and a height of 0.40 m) were used to increase *T*_*s*_ and *T*_*a*_ since July 2008. The OTCs were randomly set up and left in place year-round. There was one unwarmed plot in the vicinity of each OTC. Half of the OTCs and their paired unwarmed plots were clipped three times each year (generally in June, July and September) since 2009. Aboveground plants were clipped to approximately 1 cm in height using scissors, and the clipped aboveground biomass was removed, oven-dried at 65 °C for 48 h and weighed.

### Microclimate measurements

SM at a depth of 10 cm, *T*_*s*_ at a depth of 5 cm, *T*_*a*_ and relative humidity (RH) at a height of 15 cm were continuously monitored using weather stations (HOBO weather station, Onset Computer, Bourne, MA, USA) during the whole study period from June to September in 2013–2015. VPD was calculated using measured *T*_*a*_ and RH.

There were only 3 out of 122 days when *T*_*a*_ in the ‘W’ plots was lower than that in the ‘C’ plots in 2014 at site A (Figure S2). There were 5 and 18 out of 122 days when *T*_*a*_ in the ‘W + CL’ plots was lower than that in the ‘CL’ plots in 2014 and 2015 at site A, respectively (Figure S3). There were only 3 and 1 out of 122 days when *T*_*a*_ in the ‘W’ plots was lower than that in the ‘C’ plots in 2014 and 2015 at site B, respectively (Figure S2). There were only 1, 6 and 1 out of 122 days when *T*_*a*_ in the ‘W + CL’ plots was lower than that in the ‘CL’ plots in 2013, 2014 and 2015 at site B, respectively (Figure S3). There were 17 and 1 out of 122 days when *T*_*a*_ in the ‘W’ plots was lower than that in the ‘C’ plots in 2013 and 2014 at site C, respectively (Figure S2). There were only 2, 4 and 4 out of 122 days when *T*_*a*_ in the ‘W + CL’ plots was lower than that in the ‘CL’ plots in 2013, 2014 and 2015 at site C, respectively (Figure S3). There were only 3 out of 122 days when *T*_*s*_ in the ‘W + CL’ plots was lower than that in the ‘CL’ plots in 2014 at site A (Figure S4). There were only 9 out of 122 days when *T*_*s*_ in the ‘W’ plots was lower than that in the ‘C’ plots in 2014 at site B (Figure S5). There were only 11 out of 122 days when *T*_*s*_ in the ‘W + CL’ plots was lower than that in the ‘CL’ plots in 2014 at site B (Figure S4). There were only 7 and 5 out of 122 days when *T*_*s*_ in the ‘W’ plots was lower than that in the ‘C’ plots in 2013 and 2014 at site C, respectively (Figure S5). There were 25, 5 and 2 out of 122 days when *T*_*s*_ in the ‘W + CL’ plots was lower than that in the ‘CL’ plots in 2013, 2014 and 2015 at site C, respectively (Figure S4). However, there were no cases when both *T*_*s*_ and *T*_*a*_ were lower in the OTCs than that outside the OTCs during the three consecutive growing seasons from 2013 to 2015 at sites B and C. There were only 2 out of 122 days when both *T*_*s*_ and *T*_*a*_ were lower in the ‘W + CL’ plots than in the ‘CL’ plots at site A. These figures indicated that the OTCs were effective in elevating environmental temperature in the three alpine meadow sites in the current study.

### Vegetation indices measurements and AGB estimation

Images in a 0.50 m × 0.50 m subplot in the centre of each target plot were taken with a Tetracam Agricultural Digital Camera (ADC, Tetracam Inc., Chatsworth, CA, USA)^39^. The ADC lens was parallel to the surface at a height of approximately 1.00 m with a field of view of 0.80 m × 0.60 m and a spatial resolution of 0.40 mm. To produce the calibration parameters, images of a white Teflon plate provided by the Tetracam manufacturer were also taken by the ADC before and after each batch of target images or when the light conditions changed remarkably. The NDVI, GNDVI and SAVI were calculated with following equations (i.e., equations 1–3) using PixelWrench2 software (included with the ADC):

1 $$NDVI=\frac{{\rho }_{nir}-{\rho }_{red}}{{\rho }_{nir}+{\rho }_{red}}$$

2 $$GNDVI=\frac{{\rho }_{nir}-{\rho }_{green}}{{\rho }_{nir}+{\rho }_{green}}$$

3 $$SAVI=1.50\times \frac{{\rho }_{nir}-{\rho }_{red}}{{\rho }_{nir}+{\rho }_{red}+0.50}$$

where ${\rho }_{nir}$, ${\rho }_{red}$ and ${\rho }_{green}$ indicate the near-infrared (760–900 nm), red (630–690 nm) and green (520–600 nm) of the ADC, respectively.

A non-destructive method was used to estimate AGB, which was based on the exponential regression equation between AGB and NDVI (i.e., AGB = 10.33e^3.28NDVI^)^39^.

### GPP algorithm

A detailed description of the GPP algorithm can be found in our previous studies^54^. A concise description is only listed for this study. GPP was estimated using the following equations:

4 $$GPP=APAR\times LUE=(FPAR\times PAR)\times (LU{E}_{\max }\times {T}_{a\min scalar}\times VP{D}_{scalar})$$

where APAR is absorbed photosynthetically active radiation by the plant canopy; LUE is light-use efficiency; FPAR is the fraction of plant canopy APAR; PAR is photosynthetically active radiation; LUE_max_ is maximum LUE; *T*_aminscalar_ is temperature attenuation scalar; VPD_scalar_ is water attenuation scalar. *T*_aminscalar_ can be calculated using daily minimum *T*_*a*_ (*T*_*amin*_), and VPD_scalar_ can be calculated using daytime mean VPD^55^. FPAR can be calculated using the observed NDVI^39^.

### Statistical Analysis

For each site, a repeated-measures analysis of variance was used to estimate the main and interactive effects of experimental warming, clipping and measuring year on the *T*_*s*_, SM, *T*_*a*_, VPD, NDVI, GNDVI, SAVI, AGB and GPP (Table 1, S1). For each year, a repeated-measures analysis of variance was used to estimate the main and interactive effects of experimental warming, clipping and measuring month on the NDVI, GNDVI, SAVI, AGB and GPP (Figs 1 and 2). Linear regressions of the NDVI, GNDVI, SAVI, AGB and GPP with *T*_*s*_, SM, *T*_*a*_, VPD were conducted (Figs 3 and 4). All the statistical analyses were performed using SPSS software (version 16.0; SPSS Inc., Chicago, IL).

## Electronic supplementary material

Table S1 & Figures S1-S5
