# Supplementary material for: Clipping has stronger effects on plant production than does warming in three alpine meadow sites on the Northern Tibetan Plateau
Source: Sci Rep. 2017 Nov 27;7:16330. doi: 10.1038/s41598-017-16645-2 (PMC5703988; doi:10.1038/s41598-017-16645-2)
Supplement: Supplementary file 1 — Table S1 & Figures S1-S5 [file 41598_2017_16645_MOESM1_ESM.doc]

**Clipping has stronger effects on plant production than does warming in three alpine meadow sites on the Northern Tibetan Plateau**

Gang Fu^*^, and Zhen-Xi Shen

**Table S1** Repeated-measures analysis of variance was used to estimate the main and interactive effects of experimental warming (W), clipping (CL) and measuring year (Y) on soil temperature (*T*_s_), air temperature (*T*_a_), soil moisture (SM) and vapor pressure deficit (VPD) in the alpine meadow site A, B and C, respectively, on the Tibetan Plateau

| Model | site A | | | | site B | | | | site C | | | |
| --- | --- | --- | --- | --- | --- | --- | --- | --- | --- | --- | --- | --- |
|  | *T*_s_ | *T*_a_ | SM | VPD | *T*_s_ | *T*_a_ | SM | VPD | *T*_s_ | *T*_a_ | SM | VPD |
| W | **59.0^***^** | **569.4^***^** | **8.4^*^** | **187.2^***^** | **81.7^***^** | **141.2^***^** | **8.4^*^** | **199.3^***^** | **286.7^***^** | **391.1^***^** | **60.2^***^** | **183.0^***^** |
| CL | 0.08 | **5.44^*^** | 0.87 | 0.46 | 0.18 | 1.75 | 0.41 | **9.47^*^** | 3.53 | **15.45^**^** | 0.13 | 0.82 |
| Y | **16.0^***^** | **113.7^***^** | **31.4^***^** | **73.8^***^** | **54.9^***^** | **71.2^***^** | **25.5^***^** | **33.4^***^** | **91.0^***^** | **6.6^*^** | 0.55 | **277.9^***^** |
| W×CL | 0.19 | 7.22 | 0.81 | 0.06 | 0.04 | 1.71 | 0.08 | **5.9^*^** | 1.37 | 4.95 | 0.16 | 2.38 |
| W×Y | 0.40 | 0.68 | 0.19 | 2.56 | 0.01 | 0.28 | 0.42 | 1.98 | 2.48 | 2.08 | 0.64 | **5.6^*^** |
| CL×Y | 0.92 | **13.3^***^** | 0.91 | **9.6^**^** | 1.42 | 0.73 | 0.31 | 1.24 | 1.21 | 0.64 | 0.55 | 2.83 |
| W×CL×Y | 0.21 | 2.77 | 0.40 | 1.51 | 0.01 | 0.22 | 0.07 | 3.38 | 0.31 | 0.43 | 0.02 | 1.13 |

*, ** and *** indicates *p* < 0.05, *p* < 0.01 and *p* < 0.001, respectively

**
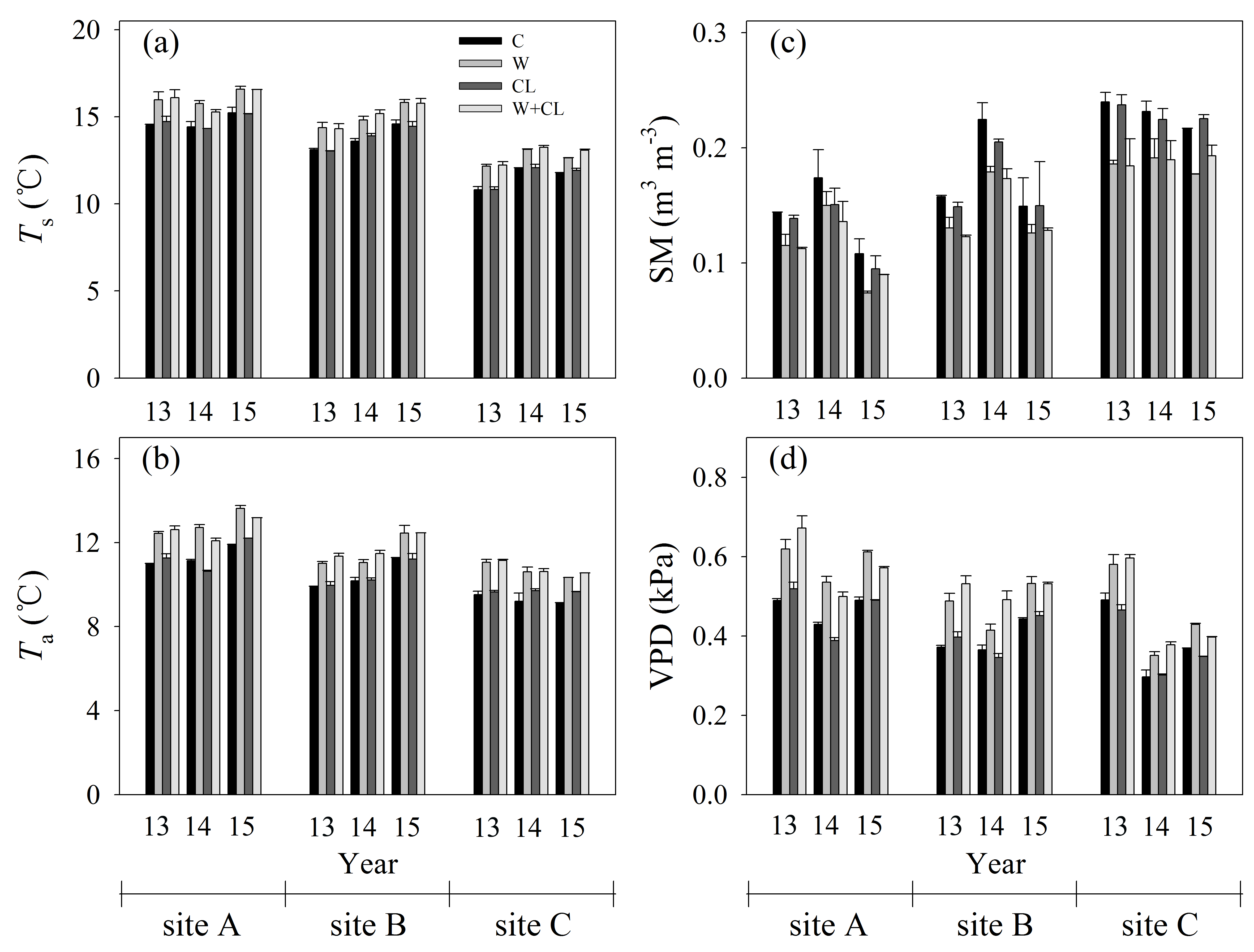
**

**Figure S1** Response of (a) soil temperature (*T*_s_), (b) air temperature (*T*_a_), (c) soil moisture (SM) and vapor pressure deficit (VPD) to experimental warming and clipping in 2013–2015 in alpine meadows at sites A, B and C on the Tibetan Plateau. C: control plots; W: warmed plots; CL: clipped plots, W+CL: warmed plus clipped plots

**
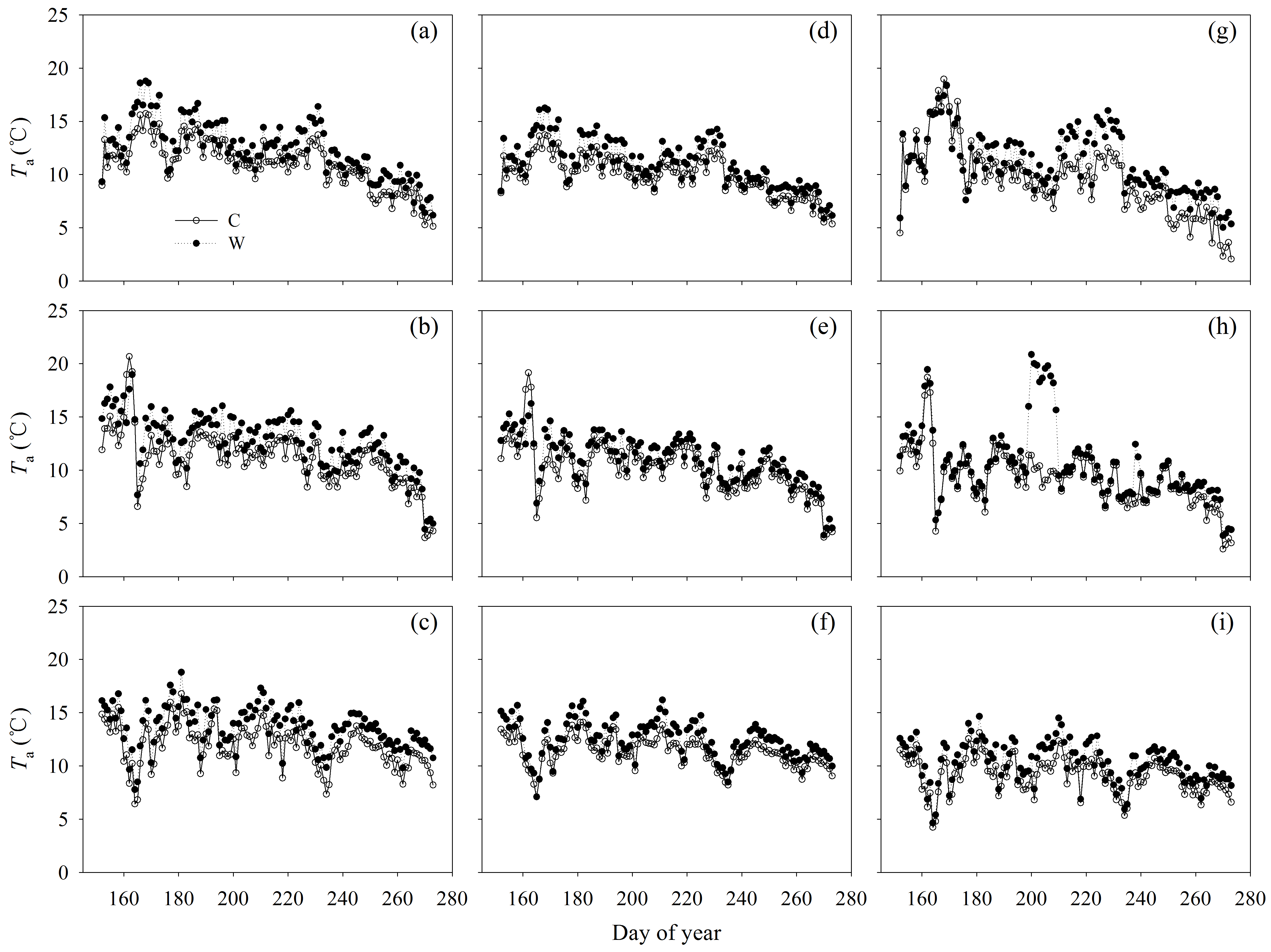
**

**Figure S2** Seasonal variation of air temperature (*T*_a_) in (a, d, g) 2013, (b, e, h) 2014 and (c, f, i) 2015 in alpine meadows at sites (a, b, c) A, (d, e, f) B and (g, h, i) C on the Tibetan Plateau. C: control plots; W: warmed plots


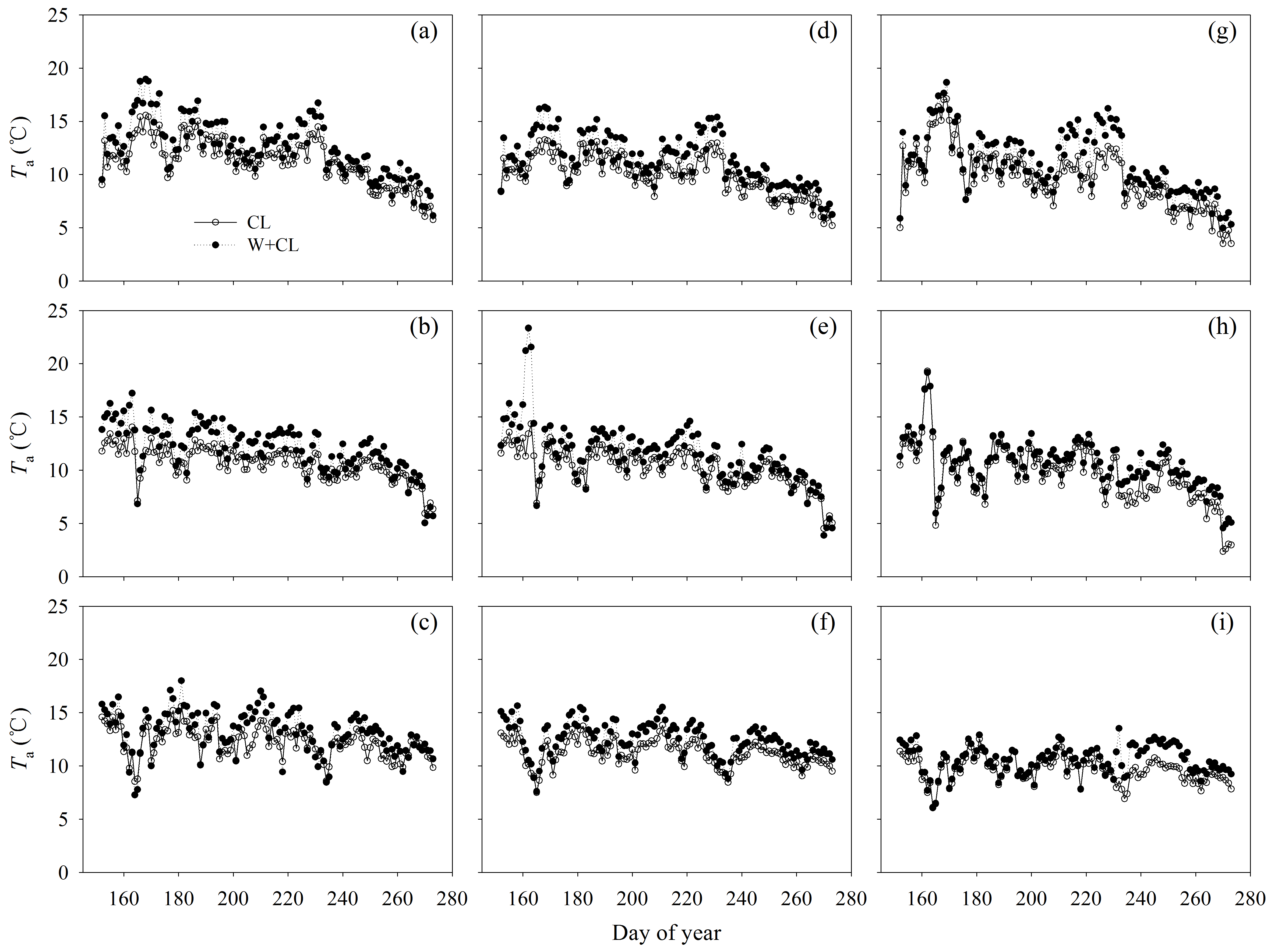


**Figure S3** Seasonal variation of air temperature (*T*_a_) in (a, d, g) 2013, (b, e, h) 2014 and (c, f, i) 2015 in alpine meadows at sites (a, b, c) A, (d, e, f) B and (g, h, i) C on the Tibetan Plateau. CL: clipped plots, W+CL: warmed plus clipped plots


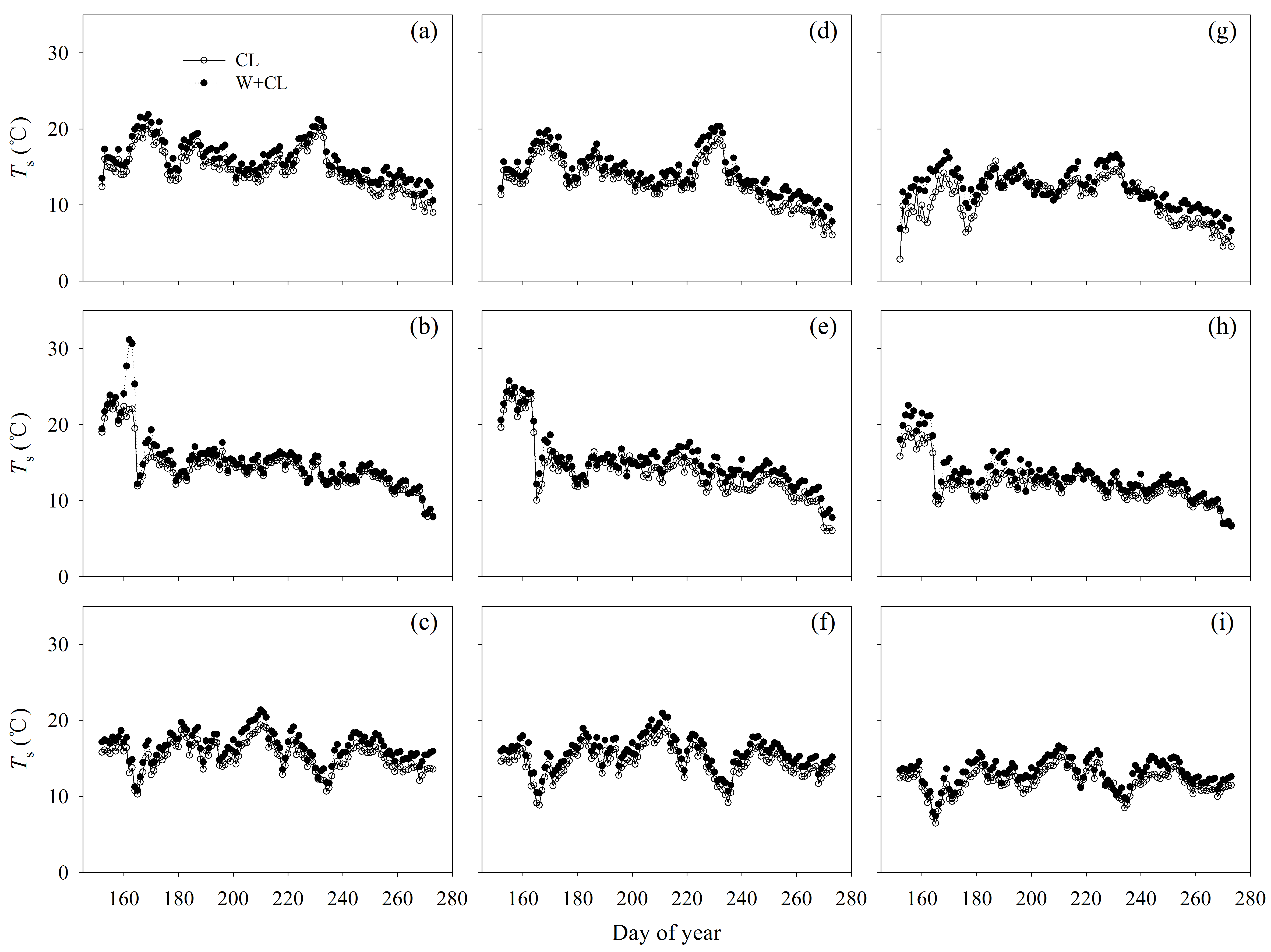


**Figure S4** Seasonal variation of soil temperature (*T*_s_) in (a, d, g) 2013, (b, e, h) 2014 and (c, f, i) 2015 in alpine meadows at sites (a, b, c) A, (d, e, f) B and (g, h, i) C on the Tibetan Plateau. CL: clipped plots, W+CL: warmed plus clipped plots


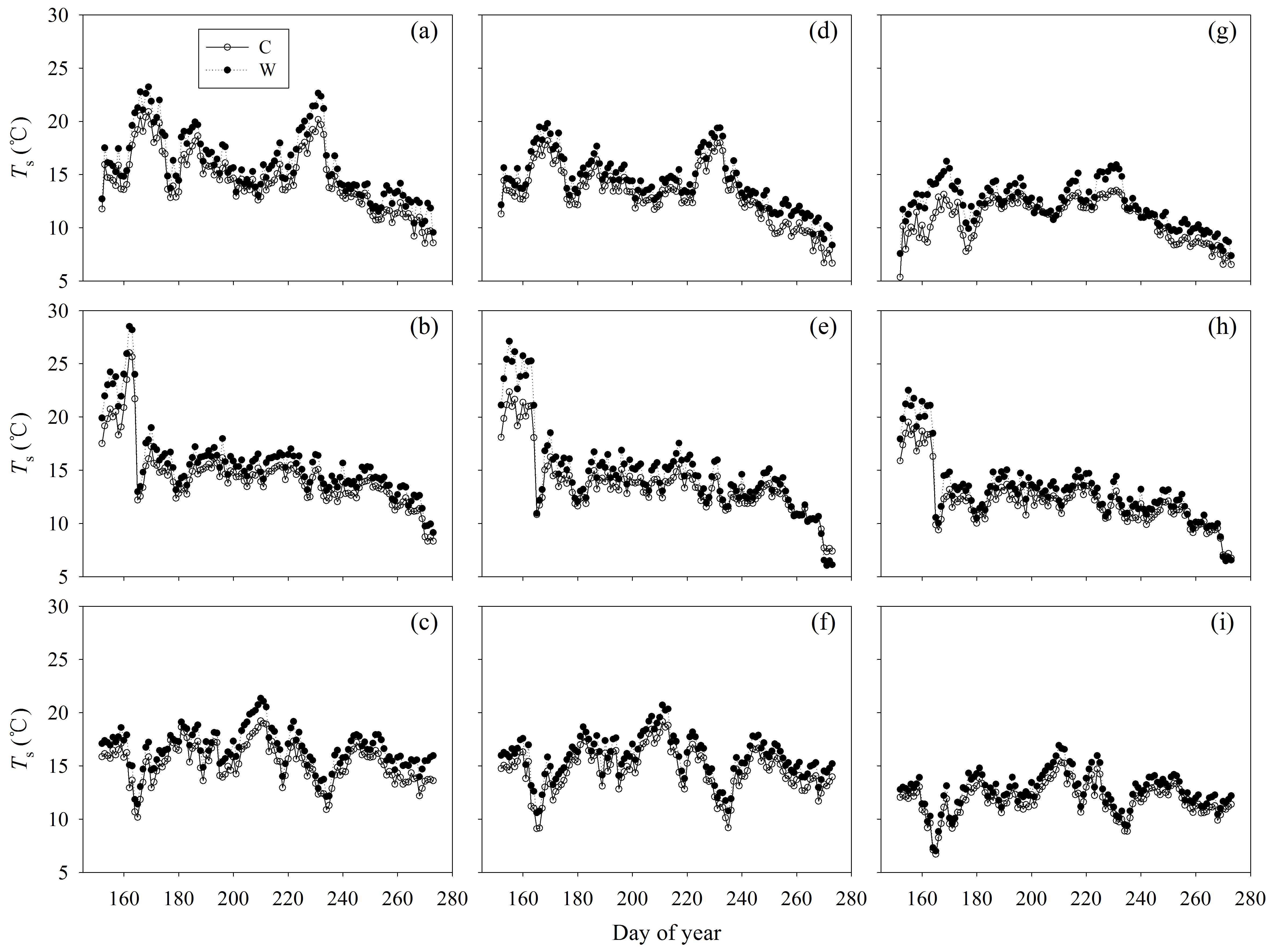


**Figure S5** Seasonal variation of soil temperature (*T*_s_) in (a, d, g) 2013, (b, e, h) 2014 and (c, f, i) 2015 in alpine meadows at sites (a, b, c) A, (d, e, f) B and (g, h, i) C on the Tibetan Plateau. C: control plots; W: warmed plots
